# WDR62 affects the progression of ovarian cancer by regulating the cell cycle

**DOI:** 10.1186/s41065-025-00444-1

**Published:** 2025-05-14

**Authors:** Yuqi Yang, Wanting Jing, Lingqi Zhang, Yuhang Zhang, Ying Shang, Ye Kuang

**Affiliations:** 1 Department of Gynecology and Obstetrics, The 2nd Affiliated Hospital of Harbin Medical University, Harbin, China; 2 Laboratory of Medical Genetics, Harbin Medical University, Harbin, China

**Keywords:** DEGs, WGCNA, Ovarian cancer, WDR62, Cell cycle, Proliferation

## Abstract

**Background:**

Ovarian Cancer (OC) is a gynecological malignant tumor with an extremely high mortality rate, seriously endangering women’s health. Due to its insidious clinical manifestations, most patients are diagnosed in the advanced stage of the disease. The currently clinically relied CA125 has limited specificity for the early diagnosis of ovarian cancer. Hence, identifying new promising biomarkers is crucial for the early screening, diagnosis, and treatment of ovarian cancer. Based on differential expression analysis, WGCNA and survival analysis, we identified a centromere-associated gene, WDR62, which is highly expressed in ovarian cancer and highly correlated with ovarian cancer, as well as the poor prognosis of ovarian cancer patients with high expression, suggesting that WDR62 may be a potential biomarker for ovarian cancer. Previous studies have shown that WDR62 is closely associated with the occurrence, development and prognosis of a variety of tumors. However, its role in ovarian cancer has not been studied in depth.

**Methods:**

Using combined TCGA and GTEx datasets from the UCSC database, along with WGCNA, and survival analysis, WDR62 was identified as a potential biomarker. GEPIA2 database, GEO database, qRT-PCR, and Western blot proved the expression of WDR62. Enrichment analysis, cell transfection, Western blots and CCK8 demonstrated the regulatory mechanism of WDR62, and the detailed mechanism of WDR62 involvement in the occurrence and development of ovarian cancer was predicted by interaction analysis and correlation analysis.

**Results:**

WDR62 was highly expressed in ovarian cancer cells compared to normal ovarian epithelial cells, both at the RNA and protein levels. Patients with high WDR62 expression had a poor survival prognosis. Upon WDR62 knockdown, the expression of cell cycle-related proteins CDK1 and *C-Myc* decreased in ovarian cancer cells, and the cell proliferative capacity was decreased. Based on bioinformatic analysis, it was hypothesized that WDR62 might mediate the JNK signaling pathway by interacting with MAPK8, thus affecting ovarian cancer progression through cell cycle regulation.

**Conclusions:**

WDR62 is overexpressed in ovarian cancer and is closely related to the prognosis of ovarian cancer patients. WDR62 promotes ovarian cancer progression by regulating the cell cycle and may influence its development through interaction with MAPK8 to mediate the JNK signaling pathway. These findings suggest that WDR62 could be a potential target for the early screening, diagnosis, and treatment of ovarian cancer.

**Supplementary Information:**

The online version contains supplementary material available at 10.1186/s41065-025-00444-1.

## Introduction

Ovarian cancer is one of the most fatal gynecological malignant tumors, often referred to as the “silent killer” of women. Due to the lack of specific symptoms and effective screening methods, 70% of patients are diagnosed at an advanced stage with metastasis. Despite surgery and adjuvant chemoradiotherapy, most patients will experience recurrence and develop chemotherapy resistance, resulting in a 5-year survival rate of only 30-40% [1–2]. Therefore, there is an urgent need to identify new potential biomarkers to improve early screening, diagnosis, and treatment of ovarian cancer.

WDR62 is a centromere-associated gene [3] located in the 19q13.12 region of the human chromosome. It has been reported to play roles in signal transduction, transcriptional regulation, cell cycle regulation, and apoptosis [4]. WDR62 is overexpressed in a variety of malignant tumors and is closely associated with tumor occurrence, development, as well as patient survival and prognosis [5]. However, to date, only one study has investigated WDR62 in ovarian cancer, demonstrating that WDR62 overexpression is associated with centrosome amplification in human ovarian cancer [6]. There is no in-depth research on the mechanisms through which WDR62 influences ovarian cancer occurrence and development.

In this study, we identified WDR62 as a potential biomarker for early screening and diagnosis of ovarian cancer and found it to be closely associated with poor patient prognosis. This was determined using data from the UCSC database, including transcriptional profiling from the TCGA and GTEx databases, WGCNA, and survival analysis. GO and GSEA enrichment analysis, along with in vitro experiments, demonstrated that WDR62 promotes ovarian cancer development through cell cycle regulation. Additionally, interaction and correlation analyses suggest that WDR62 may affect ovarian cancer progression by interacting with MAPK8 (also known as JNK1) to mediate the JNK signaling pathway.

Therefore, this study found that WDR62 may be a promising target for early screening, diagnosis, and treatment of ovarian cancer and could be used to evaluate the prognosis of ovarian cancer patients.

## Materials and methods

### Data collection

The UCSC Xena database [7] integrates data from multiple public databases such as TCGA, ICGC, and GTEx. The pan-cancer dataset “TCGA TARGET GTEx” processed by the Toil process was downloaded from the UCSC Xena database (https://xenabrowser.net, accessed on Dec 15, 2022), RNA-seq data in FPKM (G = 60499, *N* = 19131) and norm_count (G = 58582, *N* = 19120) data formats were downloaded for subsequent analysis. The GEO database [8] (http://www.ncbi.nlm.nih.gov/geo, accessed on Dec 15, 2022) contains high-throughput gene expression and genomics data from microarrays. The GSE26712 (Tumor = 185, Normal = 10) GSE12470 (Tumor = 43, Normal = 10) and GSE18520 (Tumor = 53, Normal = 10)datasets was downloaded from the GEO database for subsequent analysis.

### Data processing and differential gene screening

The “dplyr” package was used to process the pan-cancer dataset “TCGA TARGET GTEx” in the above two data formats, resulting in the extraction of the “TCGA_OV_GTEx” dataset, which includes ovarian cancer and normal ovarian tissue samples. The “DESeq2” package [9] was used to analyze the gene differential expression in the “TCGA_OV_GTEx” dataset, and the differentially expressed genes (DEGs) were screened with P value < 0.05,|log2FC|>2 as the threshold value. The above data processing was done in R (v.4.3.2).

### Weighted gene co-expression network analysis (WGCNA)

The “TCGA_OV_GTEx” dataset was analyzed using the “WGCNA” package [10], genes with the top 25% variance were selected for subsequent analysis, co-expression networks were constructed using the normalized data, and the Topological Overlap Matrix (TOM) was applied for network construction and module identification. The data were analyzed using the computational parameters minModuleSize = 30 and mergeCutHeight = 0.25. Module Membership (MM) and Gene Significance (GS) were calculated to analyze the correlation between genes, modules and clinical data, and the parameters|MM|>0.8 and|GS|>0.6 were set to screen the genes. Subsequently, hub genes were identified by intersecting differentially expressed genes (DEGs) using the Venn diagram tool(http://bioinformatics.psb.ugent.be/webtools/Venn/).

### GO and GSEA enrichment analysis

The DAVID database [11] (https://david.ncffcrf.gov/) integrates biological data and analytical tools to provide informative annotations of genes and proteins. GO (Gene Ontology) [12] is a bioinformatics tool for analyzing and annotating gene biological processes. GO enrichment analysis was performed using the DAVID database, and the functional analysis results were visualized with bubble plots created using the Sangerbox tool [13]. Referring to the gene set files provided by the MSigDB database [14] on the GSEA website, the expression matrix and phenotype files were imported, and the target gene was enriched and analyzed using the GSEA software [15]. This approach aimed to elucidate the potential mechanisms underlying ovarian cancer occurrence and development.

### Interaction network analysis

STRING (https://stringdb.org/) [16], a database for predicting and analyzing functional interactions between proteins, was used to identify functional protein-protein interactions (PPIs) for WDR62. GeneMANIA (http://genemania.org/) [17] was employed to identify gene networks containing WDR62.

### Bioinformatics analysis related to WDR62

Based on expression profiling data from the TCGA and GTEx databases, differential expression of WDR62 in various tumors and normal tissues was analyzed using the Sangerbox tool. Survival analysis was conducted with Kaplan-Meier Plotter (https://www.kmplot.com) to assess the impact of gene expression on ovarian cancer prognosis. The GEPIA2 database [18] was used to examine the differential expression of WDR62 in ovarian cancer tissues versus normal ovarian tissues and to explore correlations between WDR62 and other genes in ovarian cancer. The differential expression of WDR62 in the GSE26712 、GSE12470 and GSE18520 datasets was visualized using the “ggplot2” package [19]. The ROC curve was plotted using the “pROC” package [20] to evaluate the predictive ability of WDR62 for ovarian cancer.

### Cell line culture

Human ovarian cancer cell lines A2780, SKOV3, OVCAR3, HO8910, and human normal ovarian epithelial cell line IOSE80 were kindly donated by the School of Basic Medical Sciences, Harbin Medical University. A2780, OVCAR3, and IOSE80 cells were cultured in DMEM medium (Gibco, Shanghai, China) supplemented with 10% fetal bovine serum (Gibco, Shanghai, China) and 1% antibiotics (100 U/ml penicillin-streptomycin) (Gibco, Shanghai, China), SKOV3 and HO8910 cells were cultured in RPMI-1640 medium (Gibco, Shanghai, China) with the same FBS and 1% antibiotic concentrations. All cells were incubated in a thermostatic incubator at 37 °C with 5% CO2.

### SiRNA transfection

Before cell transfection, ovarian cancer cells in the logarithmic growth phase were plated in six-well plates and cultured for 24 h. When cell growth reached approximately 70%, transfection was performed according to the reagent instructions (Seven, Beijing, China). 30pmol of siRNA were diluted in a serum-free medium to a final volume of 50 µl. Then, 4ul of siRNA transfection reagent were added into the serum-free medium with the final volume of 50 ul, incubated for 5 min at room temperature, and then mixed with the siRNA dilution. Transfection was carried out after a 20-minute incubation. Following 48 h, protein was extracted, and transfection efficiency was verified by Western blot.

### RNA extraction and qRT-PCR

Total RNA from the cells was extracted using the Trizol method (Ambion, USA), and RNA concentration was measured with a NanoDrop spectrophotometer. cDNA was synthesized via reverse transcription using a Reverse Transcription Kit (Seven, Beijing, China). Quantitative PCR (qPCR) was performed with 2xSYBR Green qPCR MasterMix II (Seven, Beijing, China) according to the kit instructions, using cDNA as the template and GAPDH as an internal reference. Three wells were set up for each sample and repeated three times independently. Primer sequences are provided in Supplementary Table S1.

### Western blot

Western blotting was first used to determine the expression of WDR62 in ovarian cancer cell lines and normal ovarian epithelial cell lines. It was then employed to evaluate transfection efficiency and finally to measure the expression of cell cycle-related proteins. Supplementary Table S2 provides the information of the antibodies used. Cells in logarithmic growth phase were washed with PBS (Seven, Beijing, China) and lysed in RIPA buffer (Beyotime, Shanghai, China) containing 1% PMSF. The lysates were scraped, harvested, centrifuged, and the supernatant was collected to obtain the total proteins, which were quantified using the BCA assay (Beyotime, Shanghai, China), boiled and denatured. Proteins were separated by 10% SDS-PAGE gel (Epizyme, Shanghai, China) electrophoresis then transferred to a methanol-activated PVDF membrane. The membrane was blocked with Protein Free Rapid Blocking Buffer (Epizyme, Shanghai, China) for 30 min at room temperature, incubated with the primary antibody at 4℃ overnight, washed with TBST on the next day and added with the secondary antibody for 1 h at room temperature, washed the membrane and then developed under the ECL developer by adding ECL enhanced chemiluminescence reagent drop by drop.

### Cell counting kit-8

After transfection, 5000 cells per well were inoculated into 96-well plates (100ul/well), 10 µl of CCK-8 reagent (Seven, Beijing, China) was added to each well on days 0, 1, 2, and 3, and the optical density at 450 nm was measured after incubation in a thermostat incubator for 1–2 h. Statistical data were obtained and cell proliferation curves were plotted.

### Statistical analysis

Statistical analysis and data plotting were performed using SPSS 26.0, GraphPad Prism 10, and R software(version v.4.3.2). The Student’s t-test was employed to compare differences between two groups. The data was presented as the mean ± standard error of the mean (SEM) from three independent experiments. A P-value < 0.05 was considered statistically significant.

## Results

### Identifying differential genes and hub genes and screening target genes in ovarian cancer

**Fig. 1:**
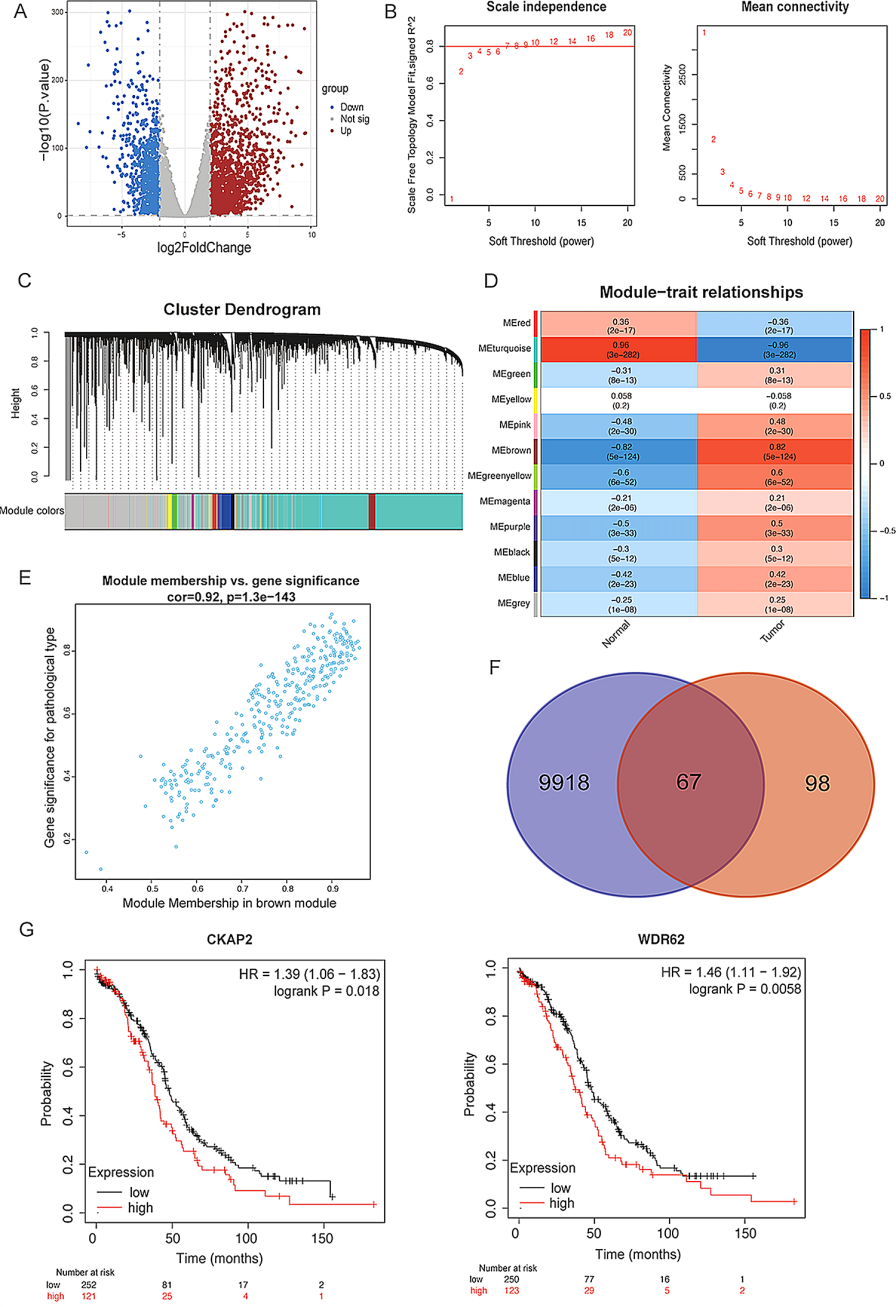
Identifying differential genes and hub genes and screening target genes in ovarian cancer. (**A**) Volcano plot of DEGs base on TCGA and GTEx databases. (**B**) Screening of soft thresholds in WGCNA. (**C**) Hierarchical clustering tree from WGCNA. (**D**) Heatmap of module-tumor correlation. (**E**) Scatter plot of Gene Significance (GS) versus Module Membership (MM) within MEbrown module. (**F**) Venn diagram depicting the intersection of DEGs and highly correlated genes by WGCNA. (**G**) Kaplan-Meier survival curves for overall survival of CKAP2 and WDR62 in ovarian cancer

Based on TCGA and GTEx datasets from the UCSC database, P value < 0.05,|log2FC|>2 was used as the threshold value to screen the differentially expressed genes (DEGs). A total of 9985 DEGs were identified, including 6635 up-regulated and 3350 down-regulated genes (Supplementary Table S3), and the volcano plot of DEGs is presented in Fig. 1A.The soft threshold β = 10 was selected in WGCNA analysis to construct a scale-free network as shown in Fig. 1B, and then the hierarchical clustering tree was constructed, and the clustering tree was cut with mergeCutHeight = 0.25 as the criterion to obtain 12 gene modules ultimately(Fig. 1C). As shown in Fig. 1D, the heatmap demonstrated the correlation between the genes modules and tumors, with the MEbrown module showing the highest correlation with ovarian cancer (*r* = 0.82, *P* < 0.05). Meanwhile, as shown in Fig. 1E, the correlation between Gene Significance (GS) and Module Membership (MM) within the MEbrown module was higher (cor = 0.92, *P* < 0.05), setting|MM|>0.8,|GS|>0.6. 165 highly correlated genes were screened, which were intersected with DEGs to obtain 67 hub genes (Supplementary Table S4) for subsequent analysis (Fig. 1F). Survival analysis of these 67 hub genes using the Kaplan-Meier Plotter website, revealed that only two genes were significantly associated with ovarian cancer survival and prognosis, WDR62 (HR = 1.46, *P* < 0.05) and CKAP2 (HR = 1.39, *P* < 0.05) (Fig. 1G). WDR62 was selected as the target gene for further analysis due to its higher prognostic hazard ratio (HR).

### WDR62 is highly expressed in tumors and correlates with poor prognosis

**Fig. 2:**
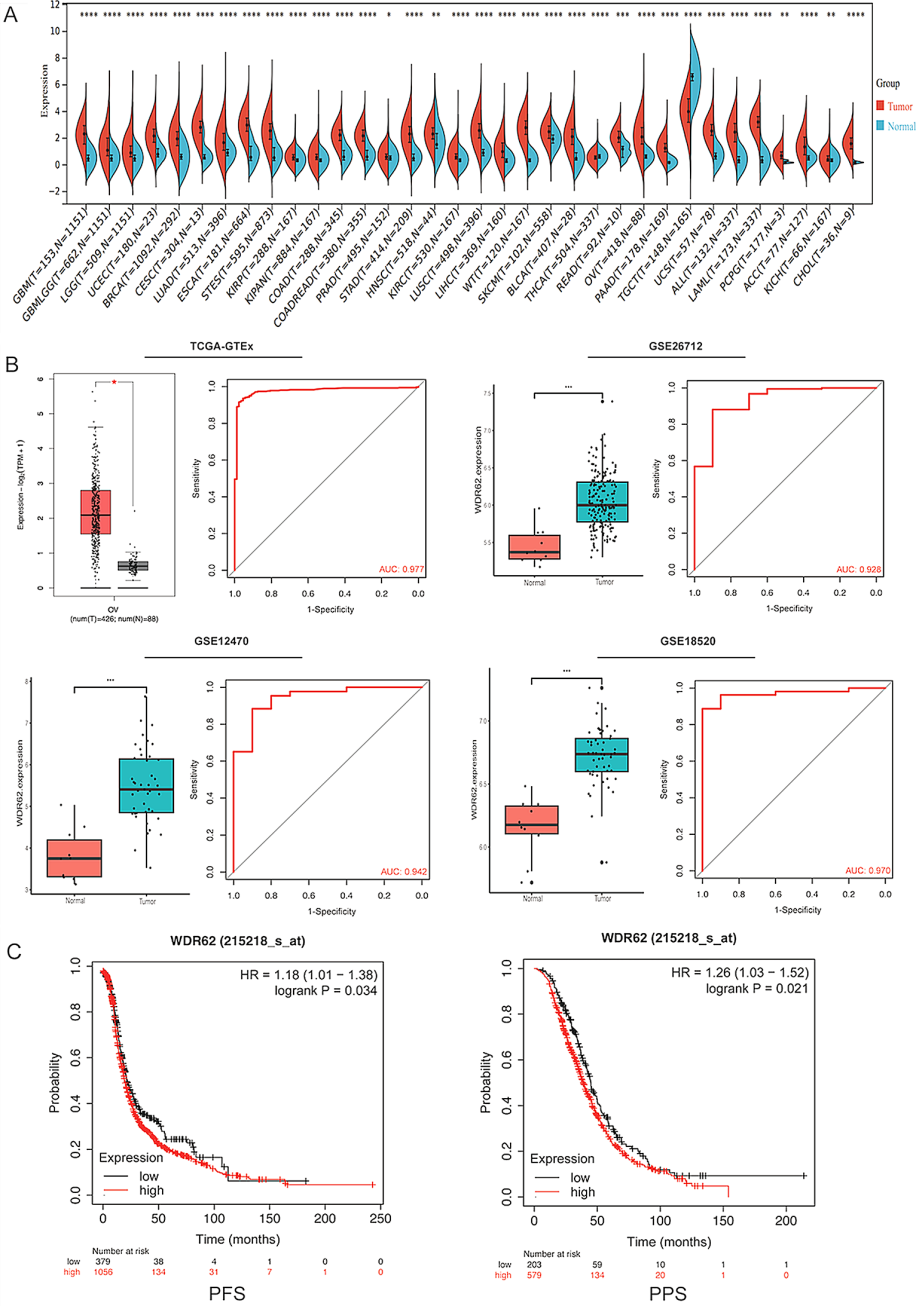
WDR62 is highly expressed in tumors and correlates with poor prognosis. (**A**) Pan-cancer differential expression analysis of WDR62 using data from the TCGA and GTEx databases. (**B**) Gene expression analysis and ROC analysis of WDR62 in TCGA-GTEx、GSE26712、GSE12470 and GSE18520 datasets. (**C**) Kaplan-Meier survival curves showing progression-free survival (PFS) and post-progression survival (PPS) of WDR62 expression in ovarian cancer patients. *P < 0.05,**P < 0.01,***P < 0.001,****P < 0.0001

Based on the data from TCGA and GTEx databases, WDR62 expression was analyzed at the pan-cancer level using the Sangerbox web tool, and the results showed that WDR62 was highly expressed in the majority of tumor types (Fig. 2A), suggesting that WDR62 functions as a broad-spectrum oncogene associated with a variety of human tumors. The analysis of the GEPIA2 database showed that WDR62 expression was significantly higher in ovarian cancer compared to normal ovarian tissue in TCGA and GTEx datasets. The ROC curve of TCGA and GTEx datasets verified the sensitivity and specificity of WDR62 as a biomarker for the diagnosis of ovarian cancer, with an area under the curve (AUC) equal to 0.977. In addition, the differential analysis of WDR62 expression in the GSE26712、GSE12470 and GSE18520 datasets further verified the high expression of WDR62 in ovarian cancer. The ROC curve corresponding to each GEO dataset further verified the sensitivity and specificity of WDR62 as a biomarker for the diagnosis of ovarian cancer, with AUC values all greater than 0.9, as shown in Fig. 2B. Additionally, Kaplan-Meier Plotter survival analysis demonstrated that high WDR62 expression was associated with poor survival prognosis of ovarian cancer patients, with elevated levels correlating with shorter the overall survival (OS), progression-free survival (PFS), and post-progression survival (PPS) (Figs. 1G and 2C).

### Functional enrichment analysis of WDR62 in ovarian cancer

**Fig. 3:**
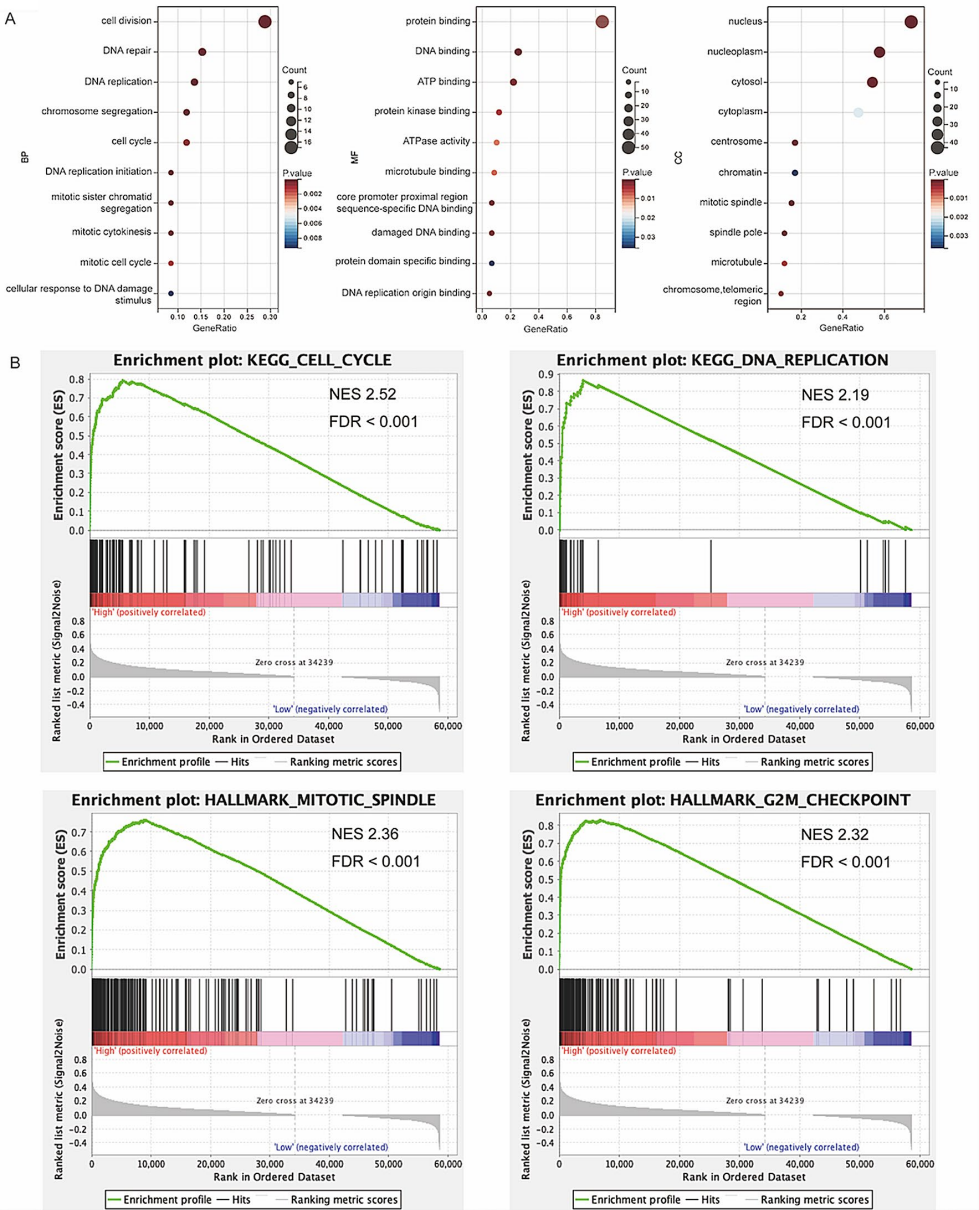
Functional enrichment analysis of WDR62 in ovarian cancer. (**A**) The bubble diagram illustrates the results of GO enrichment analysis for hub genes, including biological processes, cellular composition, and molecular functions. (**B**) GSEA enrichment analysis of WDR62 in ovarian cancer, which was divided into high-expression and low-expression groups based on the median expression levels

Since all of the 67 genes described previously are differentially expressed in ovarian cancer, and are also located in the same gene module with the highest correlation to ovarian cancer, and have a high correlation to this gene module, suggesting that these 67 genes have similar expression patterns and may be functionally related or in the same functional pathway. Therefore, GO enrichment analysis was performed on 67 hub genes using DAVID’s website, including molecular function (MF), biological process (BP), and cellular composition (CC). The results indicated that 67 hub genes was primarily involved in biological processes, such as cell division, DNA replication, chromosome segregation, and cell cycle. Cellular components affected by 67 hub genes include the nucleus, nucleoplasm, centrosome, chromatin, and mitotic spindle. Molecular functions associated with 67 hub genes include protein binding, DNA binding, ATP binding, and protein kinase binding (Fig. 3A). Additionally, the enrichment analysis of WDR62 was carried out by GSEA software based on the ovarian cancer data in the TCGA database, and according to the median expression of WDR62, it was divided into high-expression and low-expression groups. Enrichment analysis revealed that WDR62 was significantly associated with pathways such as MITOTIC_SPINDLE and G2M_CHECKPOINT in the hallmark gene set. The KEGG gene set analysis further indicated that WDR62 was mainly enriched in pathways such as CELL_CYCLE and DNA_REPLICATION (Fig. 3B). These findings suggest that overexpression of WDR62 in ovarian cancer may contribute to the progression of ovarian cancer by regulating the cell cycle.

### The expression and regulation of WDR62 in ovarian cancer were demonstrated in vitro

**Fig. 4:**
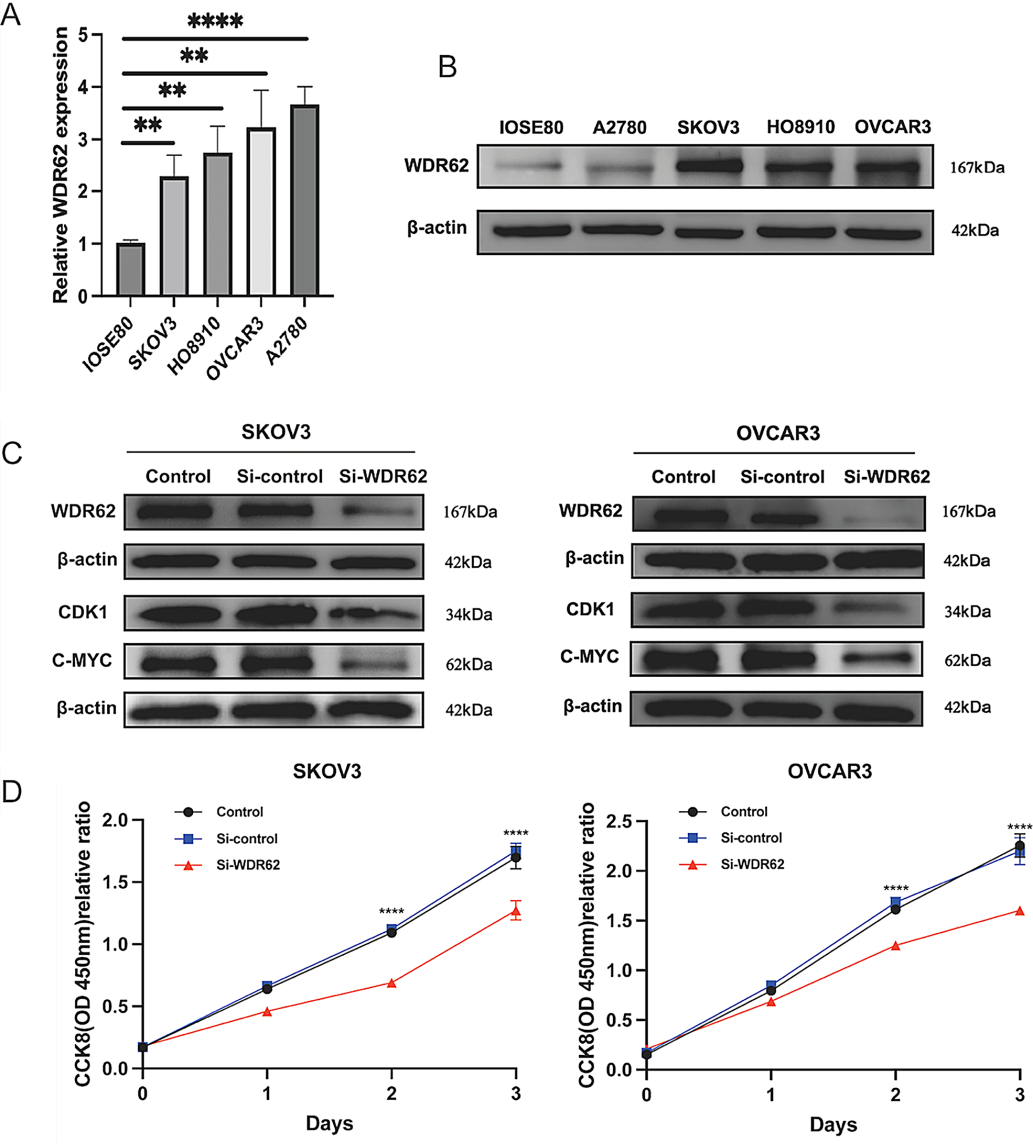
The expression and regulation of WDR62 in ovarian cancer were demonstrated in vitro. (**A**) qRT-PCR results demonstrated the differential expression of WDR62 at the RNA level in ovarian cancer cell lines compared to normal ovarian epithelial cell lines. (**B**) Western blot demonstrated the differential expression of WDR62 at the protein level between ovarian cancer cells and normal ovarian epithelial cells. (**C**) Transfection efficiency of si-WDR62 in SKOV3 and OVCAR3 cell lines. Expression of CDK1 and C-MYC after knockdown of WDR62 in SKOV3 and OVCAR3 cell lines. (**D**) CCK8 demonstrated the effect of the knockdown of WDR62 on the proliferative capacity of SKOV3 and OVCAR3 cell lines. Data were shown as mean ± SEM, *P < 0.05,**P < 0.01,***P < 0.001,****P < 0.0001

The qRT-PCR results demonstrated that the mRNA expression level of WDR62 was significantly higher in ovarian cancer cell lines (A2780, SKOV3, HO8910, and OVCAR3) compared to normal ovarian epithelial cells (IOSE80) (Fig. 4A). Similarly, as shown in Fig. 4B, Western blot analysis revealed that the WDR62 protein expression was significantly elevated in the ovarian cancer cell lines (A2780, SKOV3, HO8910, and OVCAR3) relative to normal ovarian epithelial cells (IOSE80), which aligned with the bioinformatics analysis. Given the higher WDR62 protein levels in SKOV3 and OVCAR3 cell lines, these were selected for WDR62 knockdown. The expression of WDR62 was effectively reduced by transfecting si-WDR62 into these ovarian cancer cell lines. Compared to the cells transfected with si-control, those transfected with si-WDR62 showed a significant decrease in WDR62 protein expression, confirming the success of transfection. Enrichment analysis indicated that WDR62 was primarily involved in the CELL_CYCLE and G2M_CHECKPOINT pathways. Consequently, we measured the relative expression levels of CDK1 and C-Myc in SKOV3 and OVCAR3 cells, finding that WDR62 knockdown significantly reduced the expression of both CDK1 and C-Myc, as shown in Fig. 4C. Full Western blot images including the ladder are provided in Supplementary Figure S1-3. These results indicate that WDR62 regulates the expression of cell cycle-related proteins in ovarian cancer. CCK8 results showed that the OD values of cells in the si-WDR62 group were significantly lower than those in the si-control group in SKOV3 and OVCAR3 cell lines (Fig. 4D). This confirmed that the knockdown of WDR62 reduced the proliferative ability of SKOV3 and OVCAR3 cell lines, further supporting its role in promoting ovarian cancer development through cell cycle regulation.

### WDR62 interaction and correlation analysis

**Fig. 5:**
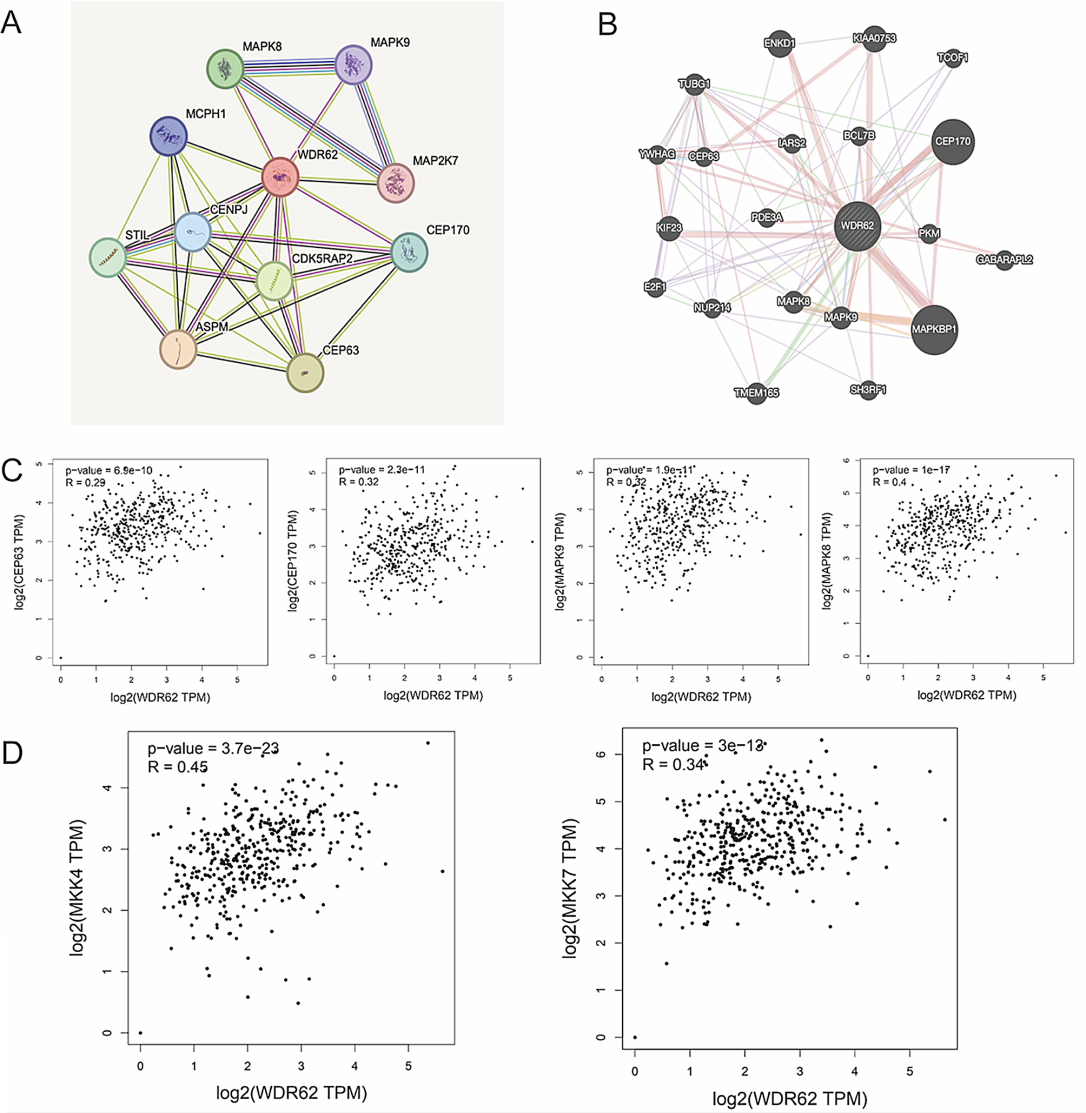
WDR62 interaction and correlation analysis. (**A**) Protein-protein interaction network of WDR62 in STRING. (**B**) Gene network of WDR62 in GeneMania, showing physical interactions or co-expression between genes. (**C**-**D**) Correlation analysis between WDR62 and MAPK8, MAPK9, CEP170, CEP63, MKK4, MKK7 in ovarian cancer using the GEPIA2 database

The interaction network of WDR62 was analyzed using STRING and GeneMANIA tools. The STRING analysis identified that WDR62 interacts with 10 proteins, including MAPK8, MAPK9, CEP170, CEP63, MCPH1, CENPJ, CDK5RAP2, ASPM, MAP2K7, and STIL (Fig. 5A). GeneMania analysis revealed that WDR62 has physical interactions or co-expressions with 20 genes, including MAPK8, MAPK9, CEP170, CEP63, MAPKBP1, ENKD1, KIAA0753, TCOF1, TUBG1, YWHAG, IARS2, BCL7B, KIF23, PDE3A, PKM, GABARAPL2, E2F1, NUP214, TMEM165, and SH3RF1 (Fig. 5B). Four genes—MAPK8, MAPK9, CEP170, and CEP63— were co-existing in the above two analyses, and the correlation between WDR62 and these four genes in ovarian cancer was analyzed by using the GEPIA2 database, the correlation between MAPK8 and WDR62 was higher (*R* = 0.4, *P* < 0.05) (Fig. 5C). Therefore, we hypothesize that WDR62 interacts with MAPK8, thereby regulating the cell cycle and promoting the development of ovarian cancer. MAPK8 is a key molecule in the JNK signaling pathway, the activation of JNK signaling is mediated by the dual phosphorylation of Thr and Tyr in the kinase activation loop by two upstream regulators, MKK4 and MKK7 [21]. Using the GEPIA2 database, we further analyzed the correlation between WDR62 and MKK4/MKK7 in ovarian cancer, as shown in Fig. 5D, WDR62 was positively correlated with MKK4 (*R* = 0.45, *P* < 0.05) and MKK7 (*R* = 0.34, *P* < 0.05) in ovarian cancer. This suggests that WDR62 might mediate the JNK signaling pathway through interacting with MAPK8 and thus regulate the cell cycle to affect the progression of ovarian cancer, but further experimental verification is needed.

## Discussion

According to statistics, there were an estimated 19,680 new cases of ovarian cancer and 12,740 related deaths in the United States in 2024 [22]. Given the significant impact of this disease, the identification of new, effective, and reliable biomarkers for early screening, diagnosis, and treatment of ovarian cancer is particularly important.

WDR62 has been reported to be overexpressed in multiple cancers and closely associated with tumor development, including bladder cancer [23], lung adenocarcinoma [24], prostate cancer [25], gastric cancer [26], colorectal cancer [27–28], and hepatocellular carcinoma [29], where it promotes tumor malignancy and correlates with a poor patient prognosis. For example, WDR62 was significantly overexpressed in LAC, and lung cancer cell proliferation decreased after WDR62 knockdown and increased after overexpression [24]. WDR62 expression was significantly elevated in gastric cancer tissues and cell lines. Knockdown of WDR62 significantly decreased gastric cancer cell proliferation and induced G2/M phase arrest of gastric cancer cells [26]. Knockdown of WDR62 significantly inhibited the proliferation, migration, and invasion of bladder cancer cells and induced apoptosis [23]. Knockdown of WDR62 also inhibited the migration and invasion of prostate cancer cells, while WDR62 overexpression increased migration and invasion [25]. In addition, knockdown of WDR62 reversed the resistance in gastric cancer cells to 5-FU, cis-diaminodichloroplatinum (CDDP), and adriamycin (ADM) [26], and elevated expression of WDR62 led to resistance to oxaliplatin and cetuximab in colorectal cancer patients [27–28]. These results suggest that WDR62 plays a significant role in the progression of various cancers, while contributes to poor prognosis and drug resistance.

In this study, we analyzed the DEGs base on combined TCGA and GTEx datasets from the UCSC database, then applied WGCNA to identify genes highly correlated with tumor progression, and then combined with survival prognostic analysis to identify WDR62 as a new potential biomarker for ovarian cancer. Pan-cancer analysis showed that WDR62 was highly expressed in a variety of tumors, and similarly, the expression level of WDR62 in ovarian cancer was significantly higher than that in normal ovaries in the TCGA-GTEx dataset, GSE26712, GSE12470, and GSE18520 datasets. ROC analysis was performed on the above datasets, the AUC values were all greater than 0.9, which further indicated that WDR62 could be used as a biomarker for ovarian cancer diagnosis. Subsequently, we confirmed the high expression of WDR62 in ovarian cancer at RNA and protein levels by qPCR and Western blot experiments, respectively. Meanwhile, the results of the survival analysis showed that high WDR62 expression was associated with poorer OS, PFS, and PPS in ovarian cancer patients. The above results indicated that WDR62 was highly expressed in ovarian cancer and was closely associated with poor prognosis of ovarian cancer patients, and WDR62 may become a new potential biomarker for ovarian cancer.

WDR62 is essential for the normal progression of mitosis and exhibits a cell cycle-dependent distribution at the spindle poles. Meanwhile, depletion of WDR62 leads to spindle orientation defects, metaphase spindle abnormalities, centrosome–spindle uncoupling, reduced centrosome integrity, and delayed mitotic completion, highlighting its critical role in mitosis and cell cycle regulation [30]. In this study, GO and GSEA enrichment analyses revealed that in ovarian cancer, the gene set associated with high WDR62 expression group was predominantly enriched in pathways such as MITOTIC_SPINDLE, G2M_CHECKPOINT, CELL_CYCLE, and DNA_REPLICATION, compared to the low-expression group. Research indicates that cell division processes are crucial for cancer progression, and imbalances in cell cycle regulation can contribute to cancer [31]. These suggest that WDR62 may affect the progression of ovarian cancer by regulating the cell cycle.

CDK1 is vital for mammalian cell cycle transition and is a crucial kinase in the G2 to M phase transition [32–34]. Additionally, C-Myc plays a significant role in cell cycle progression and cell proliferation [35]. Our results demonstrate that reducing WDR62 expression in ovarian cancer cells significantly decreases the levels of cell cycle-related proteins CDK1 and C-Myc in vitro. This suggests that WDR62 regulates the expression of cell cycle-related proteins in ovarian cancer. Meanwhile, CCK8 results showed that the knockdown of WDR62 decreased the proliferative ability of ovarian cancer cells. These results suggest that WDR62 promotes ovarian cancer development by regulating the cell cycle.

WDR62 was first identified as a scaffolding protein within the JNK signaling pathway. It features 13 repeats of the WD40 structural domain at its N-terminal, a JNK-binding domain, and six potential JNK phosphorylation sites at its C-terminal [4]. The WD40 domain is a prominent feature within proteins, well-known for mediating various protein-protein interactions [36]. In this study, we explored WDR62’s interacting proteins through STRING and GeneMania databases. Notably, MAPK8 showed the highest correlation with WDR62 in ovarian cancer (*R* = 0.4, *p* < 0.05). Therefore, we speculated that WDR62 might interact with MAPK8 in ovarian cancer.

The MAPK family regulates a variety of cellular processes by coordinating different cellular programs in response to extracellular signals [37]. The MAPK signaling pathway is activated by a cascade of protein kinases, of which MAP3K, MAPKK, and MAPK are considered to be the core cascade [38]. Three major modules exist in mammals: the ERK pathway, the JNK pathway, and the p38-MAPK pathway [4]. MAPK8, also known as JNK1, is a key molecule in the JNK signaling pathway and has been reported to play an important role in a variety of tumorigenesis. For example, MAPK8 is oncogenic in the development of gastric cancer in mice induced by N-methyl-N-nitrosourea [39]. In hepatocellular carcinoma tissues, activation of MAPK8 is increased and correlates with poor patient prognosis [40]. However, MAPK8 not only exerts pro-carcinogenic effects but may also mediate tumor suppression [41]. For example, in a mouse skin cancer model, MAPK8-deficient mice developed a greater number of papillomas [42]. In addition, MAPK8-deficient mice spontaneously produce small intestinal tumors [43]. This demonstrates that MAPK8 plays different promotional or inhibitory roles in different tumor cells or tissues. Importantly, studies have demonstrated that MAPK8 exhibits significant pro-cancer effects in ovarian cancer, with MAPK8 inhibition showing notable antitumor effects both in vivo and in vitro [44]. This suggests that MAPK8 may play a critical role in ovarian cancer, further validating that WDR62 may interact with MAPK8 thereby promoting ovarian cancer progression.

Similar to WDR62, JNK is regulated in a cell cycle-dependent manner [45]. The phosphorylation of JNK is increased in the G2/M phase [46]. Studies have shown that WDR62 can enhance JNK kinase activity [4]. Meanwhile, JNK can also mediate the phosphorylation of the C-terminal domain of WDR62 to maintain the function of the metaphase spindle structure [30]. In addition, WDR62 can also lead to multidrug resistance in gastric cancer and oxaliplatin resistance in colorectal cancer through the activation of MAPK signaling [26–27]. These suggest the close association of WDR62 with the JNK signaling pathway.

GEPIA2 database analysis showed that WDR62 is positively correlated with MKK4 and MKK7, key components of the JNK signaling pathway, in ovarian cancer. Combined with the previous discussion, MAPK8 is a key molecule in the JNK signaling pathway, and WDR62 may interact with MAPK8 to promote the progression of ovarian cancer. These results suggest that WDR62 is closely related to the JNK signaling pathway in ovarian cancer, and WDR62 may mediate the JNK signaling pathway to influence the progression of ovarian cancer by interacting with MAPK8.

To sum up, our results suggest that WDR62 may be a potential biomarker for ovarian cancer, and WDR62 is highly expressed in ovarian cancer and is closely associated with poor prognosis of ovarian cancer patients.WDR62 promotes the proliferation of ovarian cancer by regulating the cell cycle, and it is hypothesized that WDR62 may influence ovarian cancer progression by interacting with MAPK8 to mediate the JNK signaling pathway and thus modulate the cell cycle. However, there are some limitations in this study, the interaction between WDR62 and MAPK8 needs to be validated through experiments. In the future, we will focus on proving the detailed role of WDR62 in the JNK pathway. To further elucidate the detailed mechanism of WDR62 affecting ovarian cancer progression.

## Conclusion

In summary, WDR62 is overexpressed in ovarian cancer, correlates with poor patient prognosis, and promotes ovarian cancer development by regulating the cell cycle. Further analysis suggests that WDR62 may interact with MAPK8 to mediate the JNK signaling pathway, and thus regulating the cell cycle to impact ovarian cancer progression. Given its role, WDR62 holds promise as a novel target for early screening, diagnosis, and treatment of ovarian cancer, which is undoubtedly of great significance in reducing the mortality rate of ovarian cancer.

## Electronic supplementary material

Below is the link to the electronic supplementary material.

Supplementary Material 1

Supplementary Material 2

Supplementary Material 3

Supplementary Material 4

## Data Availability

Data is provided within the manuscript or supplementary information files.

## Abbreviations

OC: Ovarian Cancer
WDR62: WD Repeat Domain 62
DEGs: Differentially Expressed Genes
TCGA: The Cancer Genome Atlas
GEO: Gene Expression Omnibus
UCSC: University of California, Santa Cruz
GTEx: Genotype-Tissue Expression
WGCNA: Weighted Gene Co-expression Network Analysis
TOM: Topological Overlap Matrix
MM: Module Membership
GS: Gene Significance
GO: Gene Ontology
BP: Biological Process
CC: Cellular Composition
MF: Molecular Function
