# Supplementary figures and images for: WDR62 affects the progression of ovarian cancer by regulating the cell cycle
Source: Hereditas. 2025 May 14;162:78. doi: 10.1186/s41065-025-00444-1 (PMC12076949; doi:10.1186/s41065-025-00444-1)

Figure S1

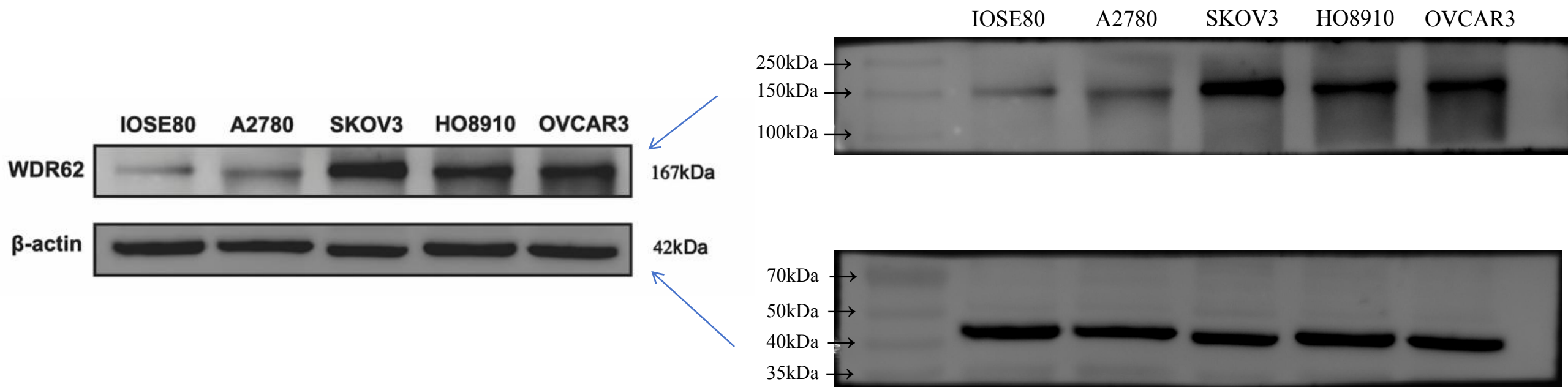

Figure S2

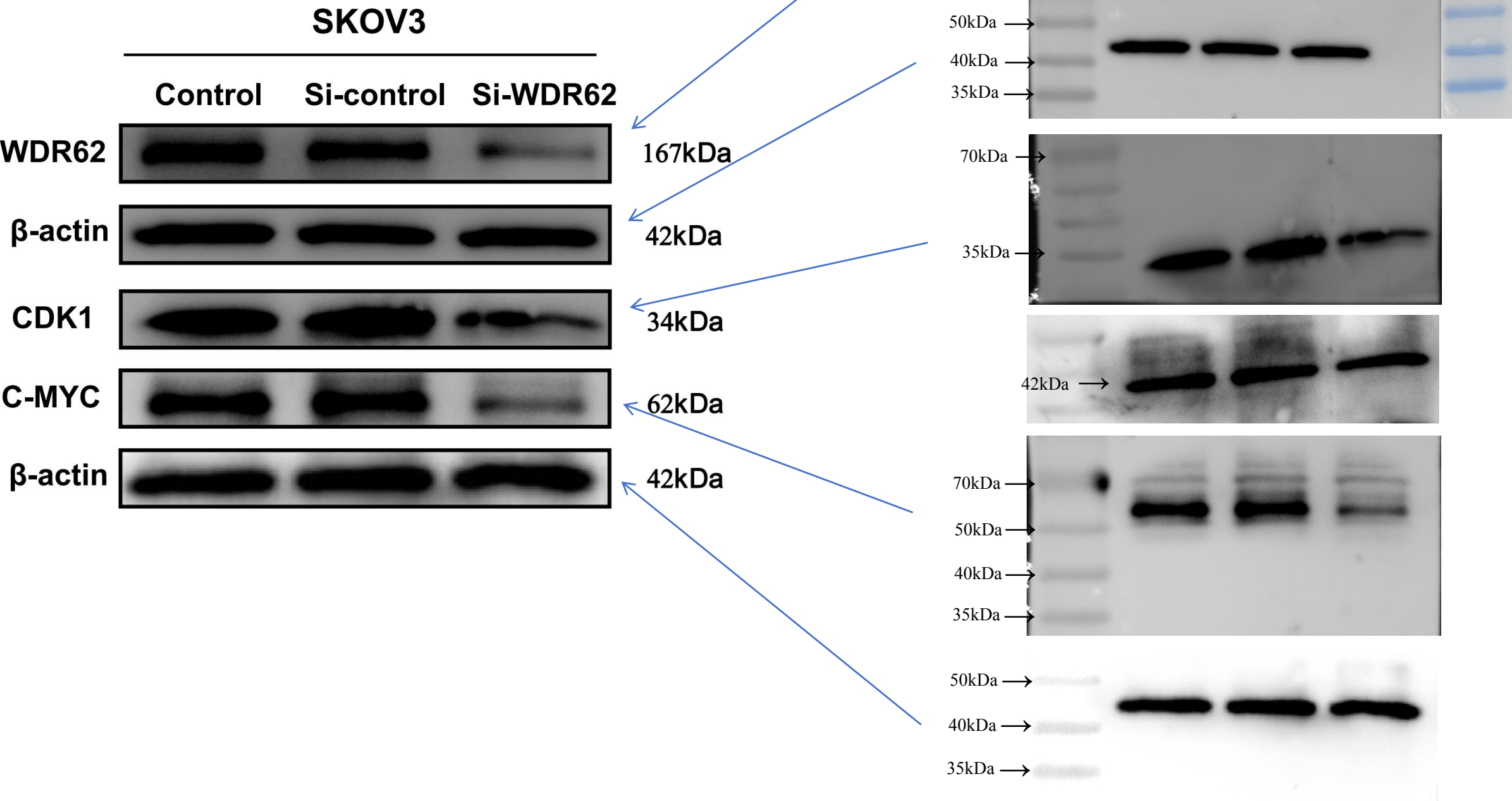

Figure S3

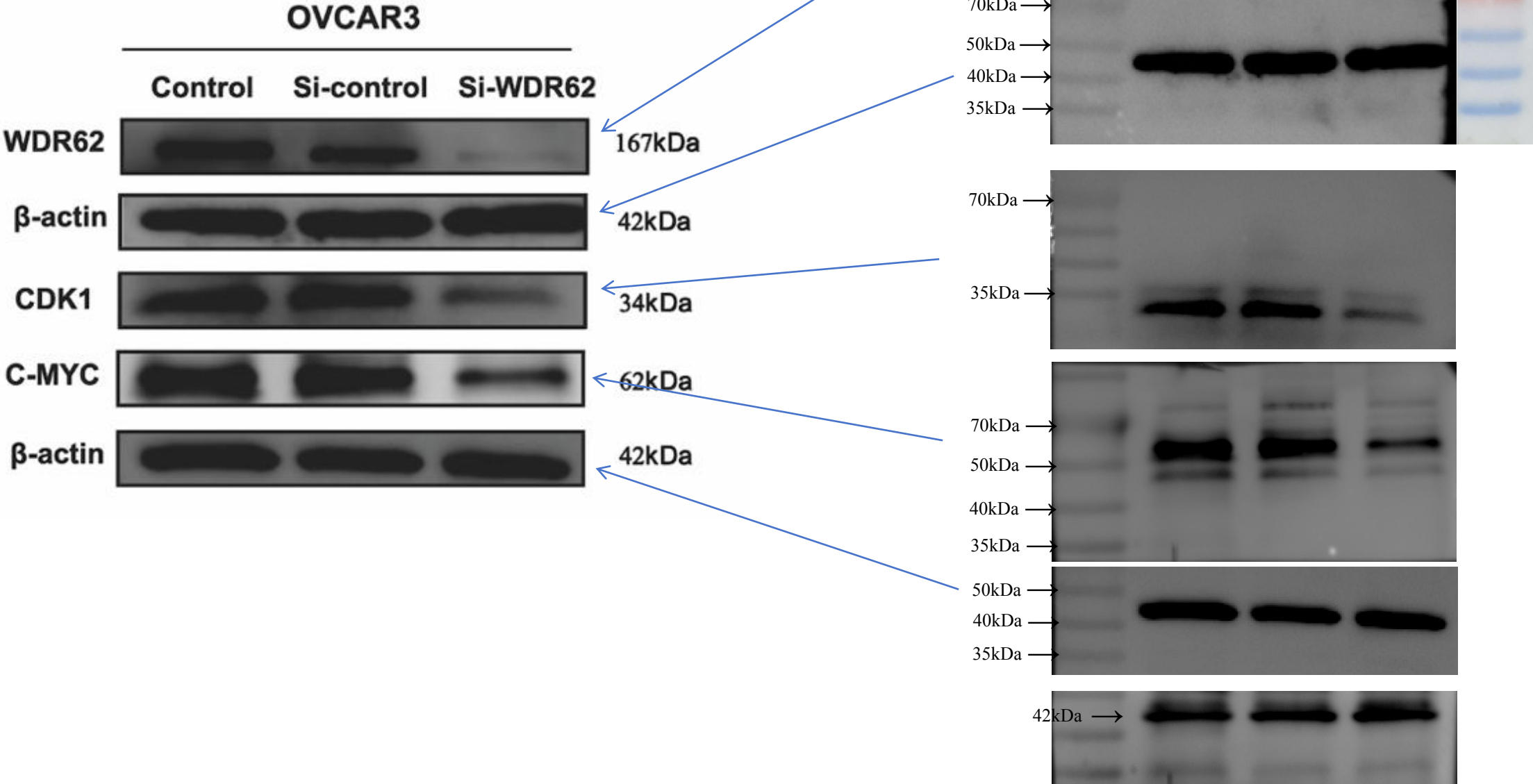

Supplement: Supplementary file 1 — Supplementary Material 1 [file 41065_2025_444_MOESM1_ESM.pdf]
