# Supplementary material for: WDR62 affects the progression of ovarian cancer by regulating the cell cycle
Source: Hereditas. 2025 May 14;162:78. doi: 10.1186/s41065-025-00444-1 (PMC12076949; doi:10.1186/s41065-025-00444-1)
Supplement: Supplementary file 2 — Supplementary Material 2 [file 41065_2025_444_MOESM2_ESM.pdf]

Table S1

Primer name	Primer sequence
WDR62F	5'- TGGCCTTCTCACCCAATATG- 3'
WDR62R	5'- GGCCACTACGATGTCTTTCT- 3'

Table S2

Name	Number	Company	Dilution
Anti-WDR62	YT7579	Immunoway	1:1000
Anti-CDK1	19532-1-AP	Proteintech	1:5000
Anti-C-myc	10828-1-AP	Proteintech	1:5000
Anti-β-actin	66009-1-Ig	Proteintech	1:25000
Anti-Mouse IgG HRP-linked	RGAM001	Proteintech	1:5000
Anti-Rabbit IgG HRP-linked	RS0002	Immunoway	1:10000
